# Supplementary material for: Arabidopsis REI-LIKE proteins activate ribosome biogenesis during cold acclimation
Source: Sci Rep. 2021 Jan 28;11:2410. doi: 10.1038/s41598-021-81610-z (PMC7844247; doi:10.1038/s41598-021-81610-z)
Supplement: Supplementary file 1 — Supplementary Information 1. [file 41598_2021_81610_MOESM1_ESM.pdf]

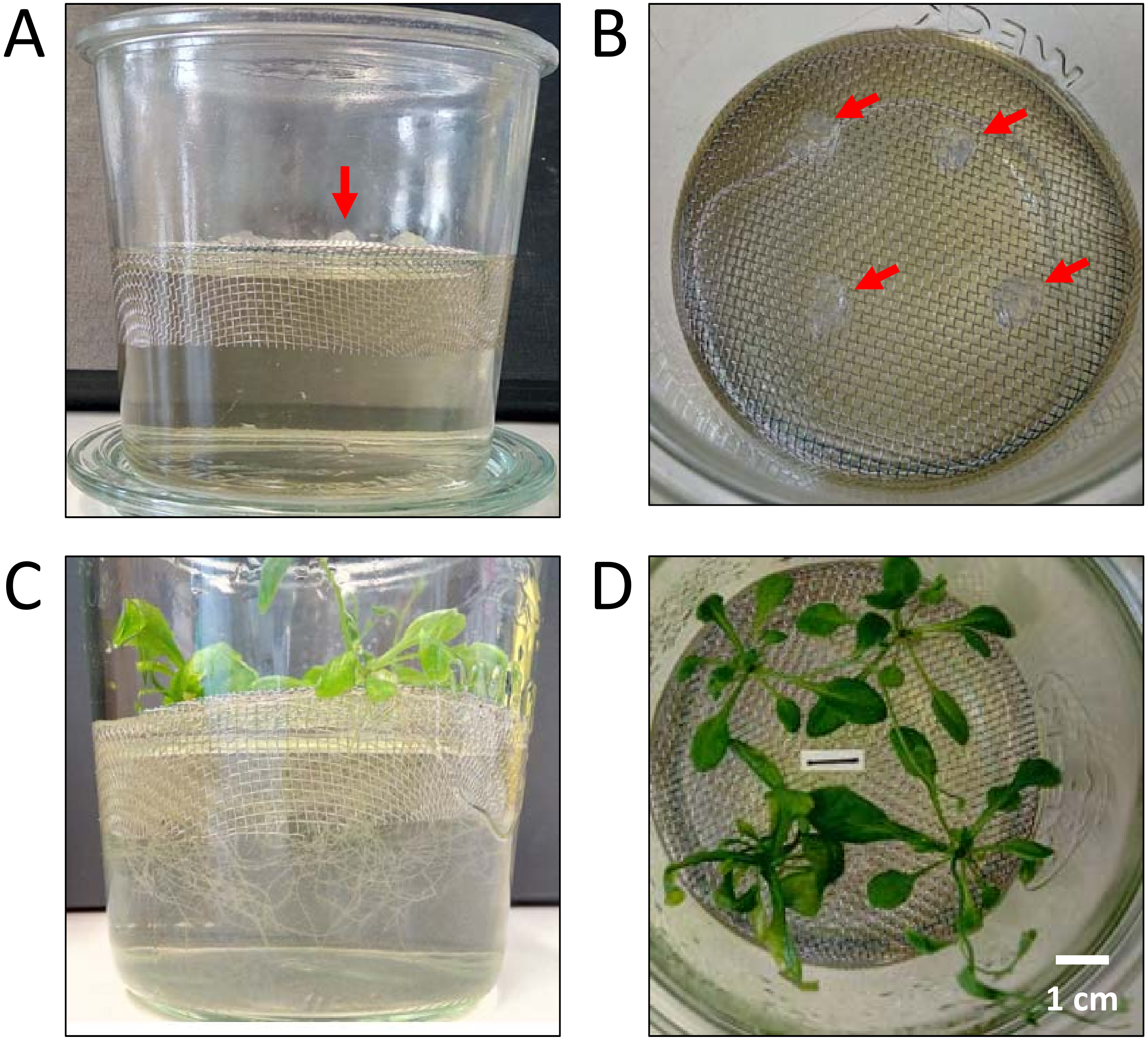
A
B
C
D
1 cm

Supplemental Figure S1. Hydroponic growth system of *Arabidopsis thaliana* Col-0 wild type and mutants. The system was feasible for temperature shift experiments in the vegetative phase and for rapid sampling of pooled material of whole root or shoot systems.

A) Assembled growth system with stainless steel mesh adjusted level with the surface of ~250 mL liquid Murashige Skoog medium with 2% sucrose [15]. One of the four agar blocks for seed germination is indicated (red arrow, view from the side).

B) Assembled growth system with stainless steel mesh adjusted level with the liquid surface. Four agar blocks for seed germination are indicated (red arrows, view from the top). Each block contains a single seed.

C) Assembled growth system with *Arabidopsis thaliana* Col-0 wild type plants at approximate ten-leaf stage after 4 weeks in 16 h/ 8 h long day conditions at 20 °C during the day and 18 °C during night (view from the side).

D) Assembled growth system with *Arabidopsis thaliana* Col-0 wild type plants at approximately ten-leaf stage (view from the top).

The clamped-on glass lids were opened and steril conditions compromised for the photographs.
