# Supplementary material for: Arabidopsis REI-LIKE proteins activate ribosome biogenesis during cold acclimation
Source: Sci Rep. 2021 Jan 28;11:2410. doi: 10.1038/s41598-021-81610-z (PMC7844247; doi:10.1038/s41598-021-81610-z)
Supplement: Supplementary file 2 — Supplementary Information 2. [file 41598_2021_81610_MOESM2_ESM.pdf]

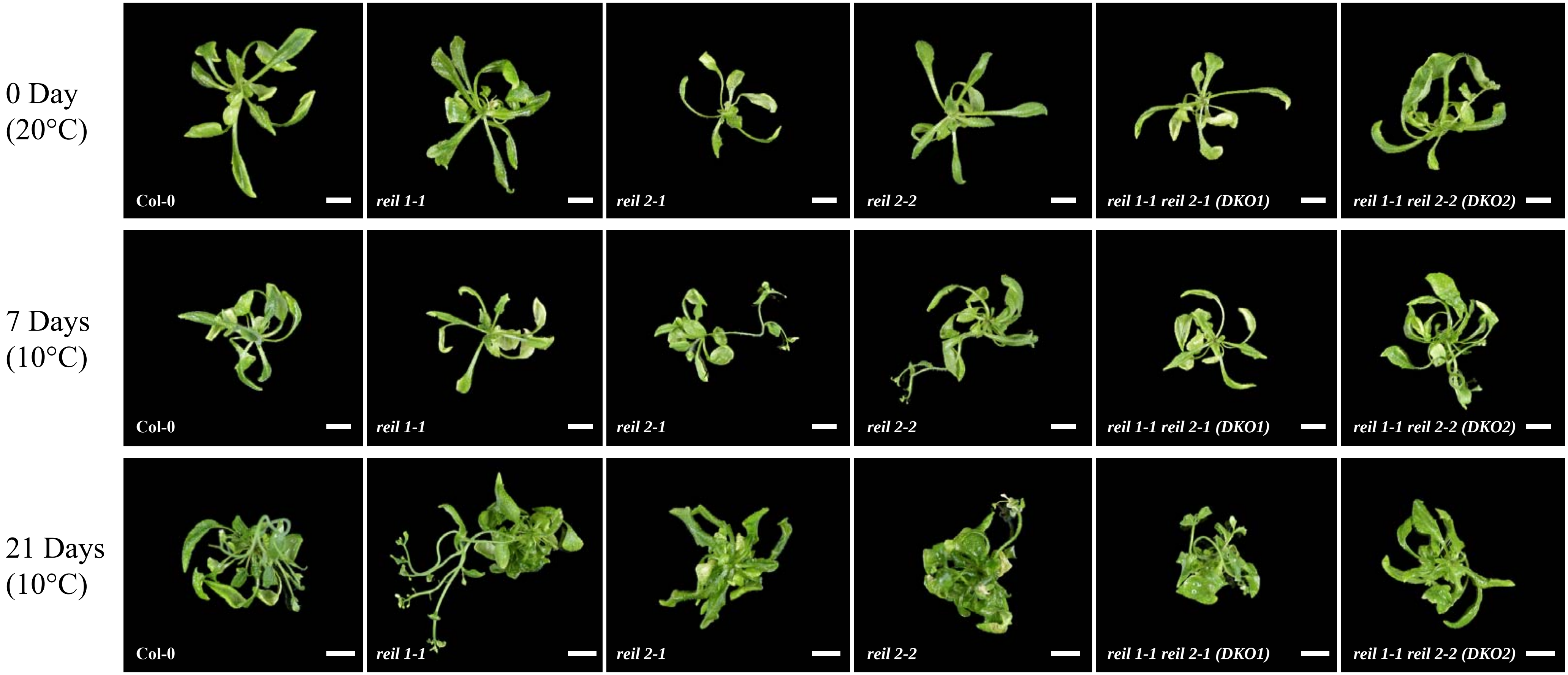
0 Day (20°C)
7 Days (10°C)
21 Days (10°C)
Col-0
reil 1-1
reil 2-1
reil 2-2
reil 1-1 reil 2-1 (DKO1)
reil 1-1 reil 2-2 (DKO2)

Supplemental Figure S2. Exemplary documentation of shoot systems of hydroponically cultivated *Arabidopsis thaliana* wild type (Col-0), of the single mutants, *reil1-1, reil2-1, reil2-2,* and of the double mutants, *reil1-1 reil2-1 (DKO1)* and *reil1-1 reil2-2 (DKO2),* before (0 day) and 7 or 21 days after shift from 20°C (day)/ 18°C (night) to 10°C (day) and 8°C (night).
Note that at 21 days after cold shift shoots and inflorescences of *reil2-1, reil2-2,* and the double mutants, *reil1-1 reil2-1 (DKO1)* and *reil1-1 reil2-2 (DKO2),* are smaller than wild type and the *reil1-1* mutant. Growth of the double mutants is not fully arrested under the conditions of this hydroponic system. Cultivation was in liquid Murashige and Skoog media with 2% sucrose (w/v) adjusted to pH 5.7 [15]. All photographs were taken separately as indicated by vertical white bars. In parts, black background was added to the single sections of the graph for a regular and centered display of the shoot systems. All bars are 1 cm and indicate the slightly varying scales of single photographs.
