# Supplementary material for: Arabidopsis REI-LIKE proteins activate ribosome biogenesis during cold acclimation
Source: Sci Rep. 2021 Jan 28;11:2410. doi: 10.1038/s41598-021-81610-z (PMC7844247; doi:10.1038/s41598-021-81610-z)
Supplement: Supplementary file 3 — Supplementary Information 3. [file 41598_2021_81610_MOESM3_ESM.pdf]

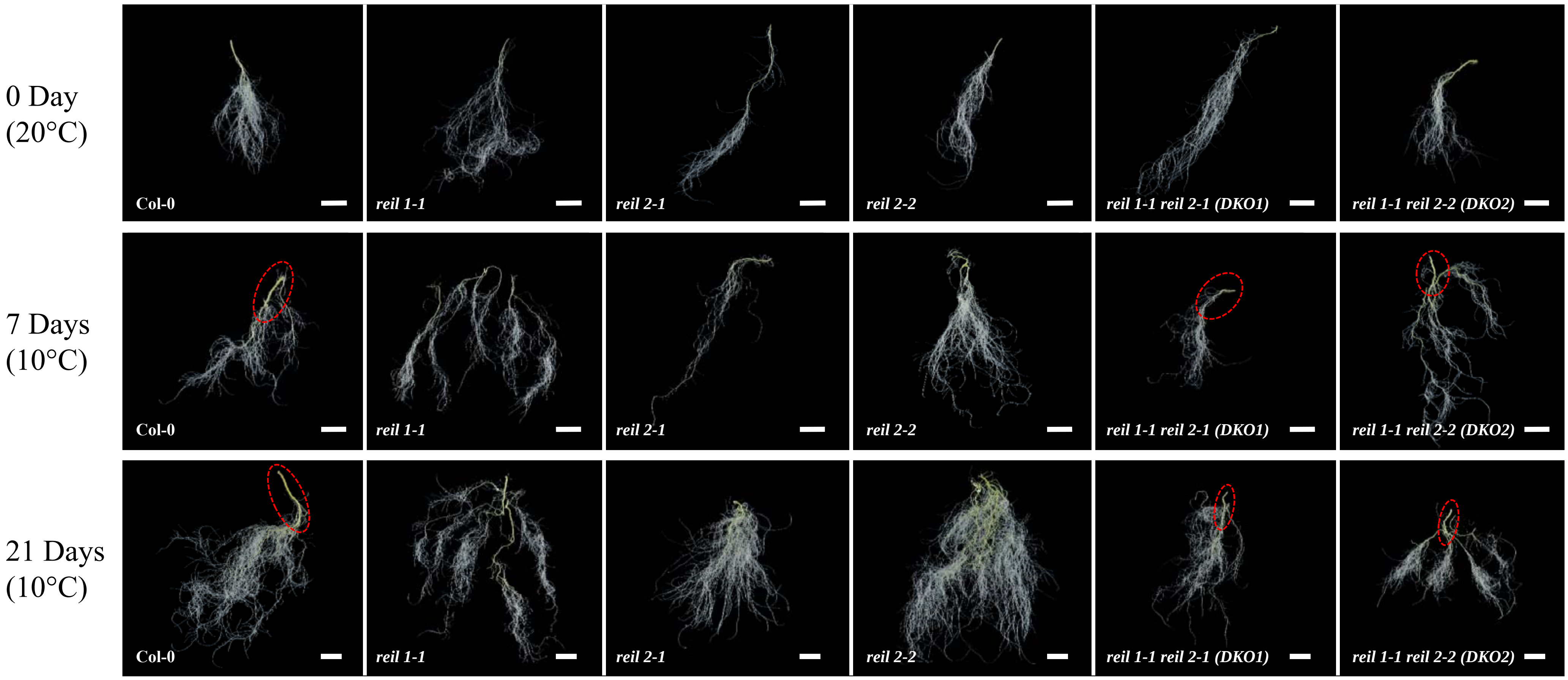
0 Day
(20°C)
7 Days
(10°C)
21 Days
(10°C)
Col-0
reil 1-1
reil 2-1
reil 2-2
reil 1-1 reil 2-1 (DKO1)
reil 1-1 reil 2-2 (DKO2)
Col-0
reil 1-1
reil 2-1
reil 2-2
reil 1-1 reil 2-1 (DKO1)
reil 1-1 reil 2-2 (DKO2)
Col-0
reil 1-1
reil 2-1
reil 2-2
reil 1-1 reil 2-1 (DKO1)
reil 1-1 reil 2-2 (DKO2)

**Supplemental Figure S3.** Exemplary documentation of hydroponic root systems of *Arabidopsis thaliana* wild type (Col-0), of the single mutants, *reil1-1*, *reil2-1*, *reil2-2*, and of the double mutants, *reil1-1 reil2-1* (*DKO1*) and *reil1-1 reil2-2* (*DKO2*), before (0 day) and 7 or 21 days after shift from 20°C (day)/ 18°C (night) to 10°C (day) and 8°C (night).
Roots were cut at the hypocotyl to root transition. Root systems of single plants were carefully prepared with minimal wounding from joined cultivations of four plants in single containers. Preparation of complete root systems from single plants was not possible. Photographs may therefore show in minor parts incomplete root systems. Note the shortened primary root of both double mutants at 7 and 21 days after cold shift compared to wild type. The respective primary root sections are indicated by red circles. At 21 days after cold shift, the double mutants and *reil1-1* appeared less branched indicative of an possibly altered root branching pattern. Cultivation was in liquid Murashige and Skoog media with 2% sucrose (w/v) adjusted to pH 5.7 [15]. All photographs were taken separately as indicated by vertical white bars. In parts, black background was added to the single section of the graph for a regular and centered display of the root systems. All bars are 1 cm and indicate the slightly varying scales of single photographs.
