# Supplementary material for: Arabidopsis REI-LIKE proteins activate ribosome biogenesis during cold acclimation
Source: Sci Rep. 2021 Jan 28;11:2410. doi: 10.1038/s41598-021-81610-z (PMC7844247; doi:10.1038/s41598-021-81610-z)
Supplement: Supplementary file 4 — Supplementary Information 4. [file 41598_2021_81610_MOESM4_ESM.pdf]

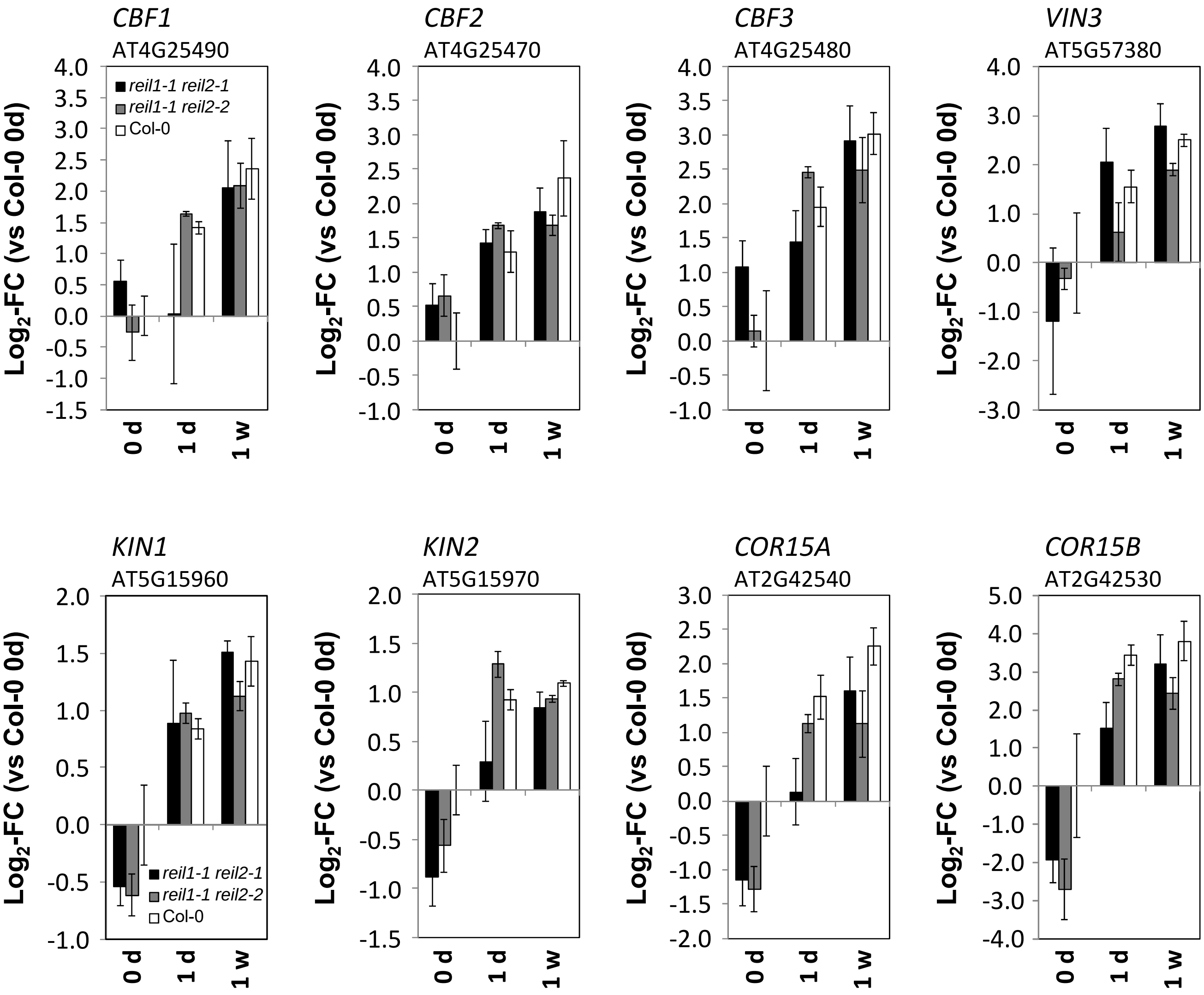

CBF1
AT4G25490
CBF2
AT4G25470
CBF3
AT4G25480
VIN3
AT5G57380
KIN1
AT5G15960
KIN2
AT5G15970
COR15A
AT2G42540
COR15B
AT2G42530
reil1-1 reil2-1
reil1-1 reil2-2
Col-0
Log2-FC (vs Col-0 0d)
0 d
1 d
1 w

Supplemental Figure S4.

Differential expression of selected cold responsive genes in the roots of Col-0, and the *reil1-1 reil2-1* and *reil1-1 reil2-2* double mutants in the non-acclimated state (0 day, 20°C) and shifted to 10°C cold for 1 day or 1 week.

Differential gene expression is determined relative to non-acclimated Col-0 at optimal temperature 20°C. The selected genes belong to GO term GO:0009409, response to cold, and in part to GO term GO:0009631, cold acclimation. Note that the marker genes indicate deregulation of cold marker gene expression in non-cold acclimated *reil* double mutant roots. Analysis of variance of each of the examples indicates a significant temperature effect ($P < 0.001$, 2 way-ANOVA) and no significant interaction with the mutant effect. The mutant effect is significant ($P < 0.05$, 2 way-ANOVA) in the cases of *KIN2*, *COR15A*, and *COR15B*.
