# Supplementary material for: Arabidopsis REI-LIKE proteins activate ribosome biogenesis during cold acclimation
Source: Sci Rep. 2021 Jan 28;11:2410. doi: 10.1038/s41598-021-81610-z (PMC7844247; doi:10.1038/s41598-021-81610-z)
Supplement: Supplementary file 5 — Supplementary Information 5. [file 41598_2021_81610_MOESM5_ESM.pdf]

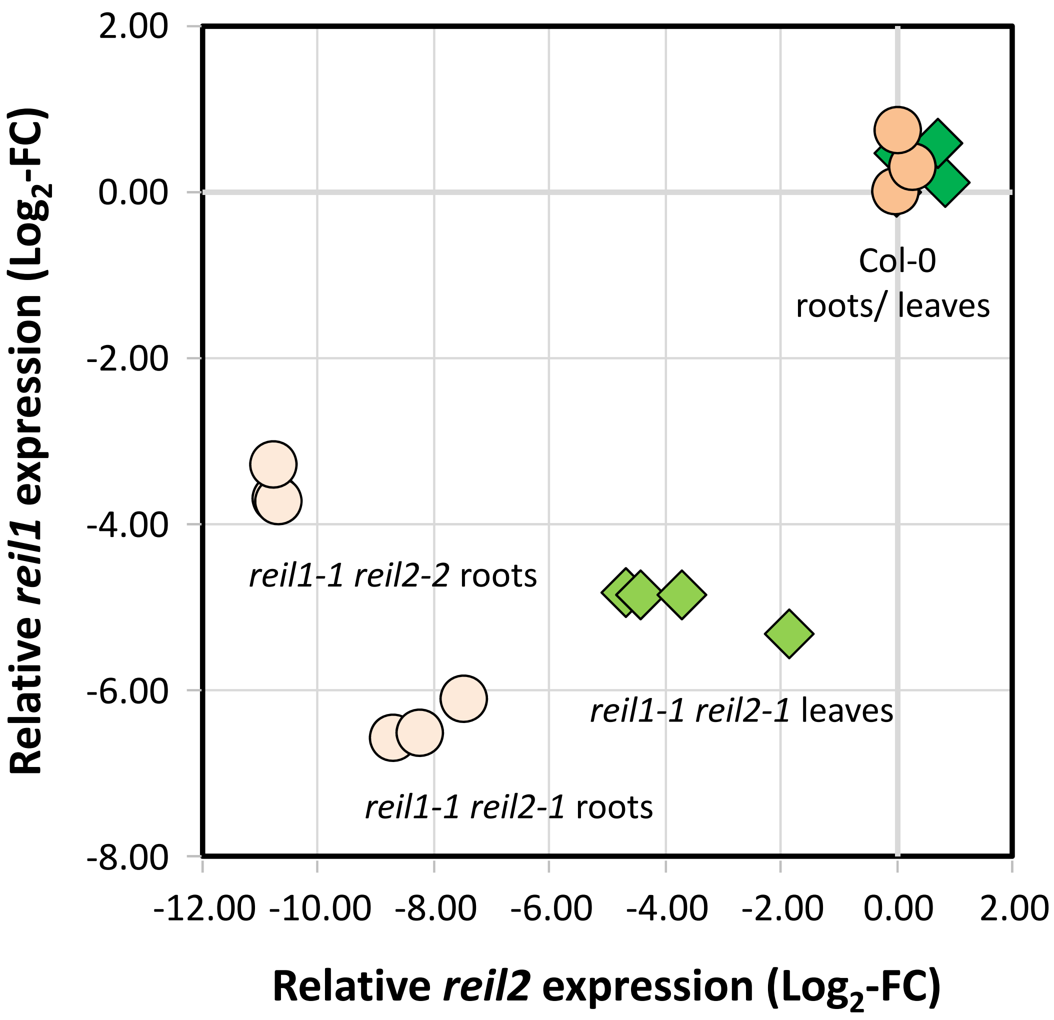

Relative *reil1* expression (Log2-FC)
Relative *reil2* expression (Log2-FC)
2.00
0.00
-2.00
-4.00
-6.00
-8.00
-12.00
-10.00
-8.00
-6.00
-4.00
-2.00
0.00
2.00
Col-0
roots/ leaves
reil1-1 reil2-2 roots
reil1-1 reil2-1 leaves
reil1-1 reil2-1 roots

Supplemental Figure S5. Reduced transcript levels of the *reil 1* and *reil2* genes in the *reil1-1 reil2-1* and *reil1-1 reil2-2* double mutants.
Root transcript data are of this study (**Supplemental Table S2**). Leaf transcript data are from soil-grown rosette plants of *reil1-1 reil2-1* from a previous study (Supplemental Table S2 of Beine-Golovchuk et al., 2018). Log_2-transformed transcript data are normalized to the non-acclimated Col-0 wild type, i.e. log_2-fold changes (FC), of each transcriptome experiment, respectively. Roots (circles), leaves (diamonds).
