# Supplementary material for: Arabidopsis REI-LIKE proteins activate ribosome biogenesis during cold acclimation
Source: Sci Rep. 2021 Jan 28;11:2410. doi: 10.1038/s41598-021-81610-z (PMC7844247; doi:10.1038/s41598-021-81610-z)
Supplement: Supplementary file 6 — Supplementary Information 6. [file 41598_2021_81610_MOESM6_ESM.pdf]

A

GO (Onthology)		Description	Genes	Col-0			*reil1-1 reil2-1*			*reil1-1 reil2-2*		
				0 d	1 d	1 w	0 d	1 d	1 w	0 d	1 d	1 w
GO:0048589	P	developmental growth	211		-0.08 *	-0.21 *	-0.18 *	-0.13 *	-0.20 *	-0.11 *	-0.12 *	-0.11 *
GO:0048468	P	cell development	158		-0.06	-0.22 *	-0.15 *	-0.16 *	-0.24 *	-0.12 *	-0.16 *	-0.13 *
GO:0021700	P	developmental maturation	47		-0.20 *	-0.40 *	-0.23 *	-0.29 *	-0.38 *	-0.28 *	-0.36 *	-0.26 *
GO:0048469	P	cell maturation	36		-0.23 *	-0.47 *	-0.30 *	-0.31 *	-0.45 *	-0.35 *	-0.39 *	-0.30 *
GO:0010015	P	root morphogenesis	91		-0.11	-0.28 *	-0.17 *	-0.18 *	-0.28 *	-0.17 *	-0.20 *	-0.15 *
GO:0010053	P	root epidermal cell differentiation	49		-0.21 *	-0.45 *	-0.29 *	-0.33 *	-0.45 *	-0.33 *	-0.38 *	-0.26 *
GO:0048765	P	root hair cell differentiation	36		-0.23 *	-0.47 *	-0.30 *	-0.31 *	-0.45 *	-0.35 *	-0.39 *	-0.30 *
GO:0010054	P	trichoblast differentiation	43		-0.19 *	-0.45 *	-0.29 *	-0.30 *	-0.45 *	-0.35 *	-0.37 *	-0.25 *
GO:0048764	P	trichoblast maturation	36		-0.23 *	-0.47 *	-0.30 *	-0.31 *	-0.45 *	-0.35 *	-0.39 *	-0.30 *
GO:0071554	P	cell wall organization or biogenesis	233		-0.22 *	-0.30 *	-0.20 *	-0.24 *	-0.29 *	-0.13 *	-0.10 *	-0.15 *
GO:0016757	F	transferase activity, transferring glycosyl	484		-0.10 *	-0.12 *	-0.12 *	-0.10 *	-0.10 *	-0.08 *	-0.08 *	-0.09 *
GO:0016758	F	transferase activity, transferring hexosyl	303		-0.13 *	-0.13 *	-0.13 *	-0.12 *	-0.12 *	-0.11 *	-0.11 *	-0.13 *

B

GO (Onthology)		Description	Genes	Col-0			*reil1-1 reil2-1*			*reil1-1 reil2-2*		
				0 d	1 d	1 w	0 d	1 d	1 w	0 d	1 d	1 w
GO:0009058	P	biosynthetic process	3716		0.04 *	0.01 *	-0.05 *	0.05 *	0.02 *	0.01 *	0.03 *	0.01 *
GO:0009059	P	macromolecule biosynthetic process	2505		0.06 *	0.03 *	-0.04 *	0.07 *	0.04 *	0.02 *	0.05 *	0.03 *
GO:0034645	P	cellular macromolecule biosynthetic pro	2483		0.07 *	0.03 *	-0.04 *	0.07 *	0.04 *	0.02 *	0.05 *	0.04 *
GO:0044249	P	cellular biosynthetic process	3536		0.05 *	0.02 *	-0.05 *	0.06 *	0.02 *	0.01 *	0.04 *	0.02 *
GO:0016137	P	glycoside metabolic process	86		0.01	-0.04	0.17 *	0.25 *	0.17 *	0.24 *	0.21 *	0.13 *
GO:0030145	F	manganese ion binding	30		0.04	0.00	-0.56 *	0.29 *	0.27 *	-0.41 *	0.47 *	0.22 *

Supplemental Figure S6. Functional enrichment analyses of differential gene expression in the roots of Col-0, and the *reil1-1 reil2-1* and *reil1-1 reil2-2* double mutants in the non-acclimated state (0 day, 20°C) and shifted to 10°C cold for 1 day or 1 week. Differential gene expression is determined relative to non-acclimated Col-0 at optimized temperature 20°C.

(A) Mean log_2-FCs of developmental, cell wall, and root related GO terms.

(B) Mean log_2-FCs of large biosynthesis related and miscellaneous GO terms.

(C = cellular component, P = biological process, F = molecular function). Significant positive or negative functional enrichments, i.e. FDR-adjusted $P < 0.05$, are indicated by asterisks. The heat map color scale is log_2-FC +1.0 (red) to -1.0 (blue). Mean log_2-FC, z-scores, and FDR-adjusted *P*-values of gene sets from 2145 GO terms are calculated by parametric analysis of gene set enrichment (PAGE) [19]. The full data set is listed in Supplemental Table S3.
