# Supplementary material for: Arabidopsis REI-LIKE proteins activate ribosome biogenesis during cold acclimation
Source: Sci Rep. 2021 Jan 28;11:2410. doi: 10.1038/s41598-021-81610-z (PMC7844247; doi:10.1038/s41598-021-81610-z)
Supplement: Supplementary file 7 — Supplementary Information 7. [file 41598_2021_81610_MOESM7_ESM.pdf]

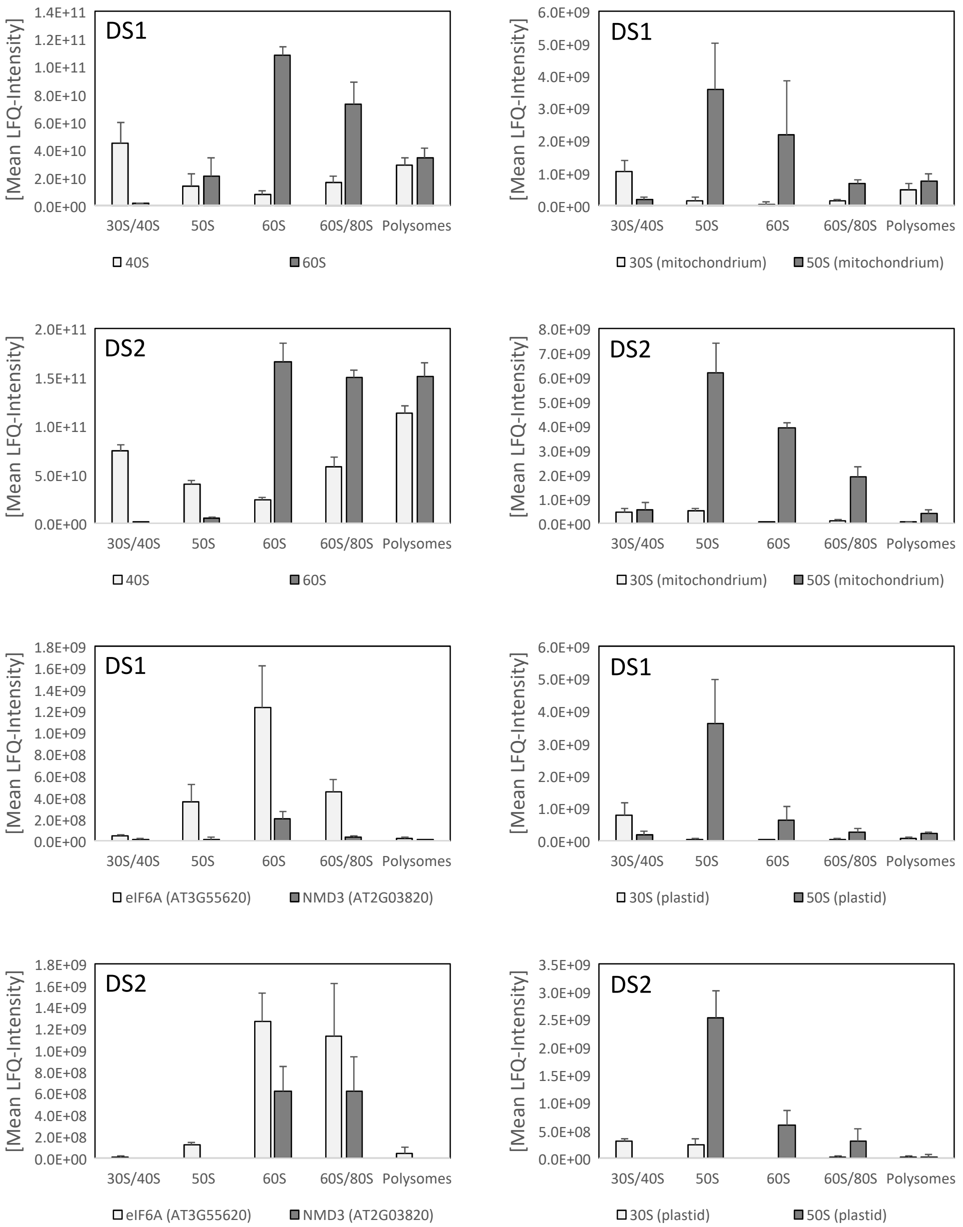
DS1
DS2
[Mean LFQ-Intensity]
30S/40S
50S
60S
60S/80S
Polysomes
40S
60S
30S (mitochondrium)
50S (mitochondrium)
eIF6A (AT3G55620)
NMD3 (AT2G03820)
30S (plastid)
50S (plastid)
0.0E+00
2.0E+10
4.0E+10
6.0E+10
8.0E+10
1.0E+11
1.2E+11
1.4E+11
5.0E+10
1.5E+11
2.0E+11
1.0E+09
2.0E+09
3.0E+09
4.0E+09
5.0E+09
6.0E+09
7.0E+09
8.0E+09
2.0E+08
4.0E+08
6.0E+08
8.0E+08
1.2E+09
1.4E+09
1.6E+09
1.8E+09
5.0E+08
1.5E+09
2.5E+09
3.5E+09

Supplemental Figure S7. Proteomic characterization of ribosome preparations obtained from root material by sucrose density gradient fractionation experiments DS1 and DS2, i.e. the root subset of Pride repository data set PXD016292. Ribosome complexes are represented by sums of LFQ-intensities of all detected RPs of 40S, 60S, and mitochondrial or plastid 30S and 50S subunits within each fraction. Non-translating immature 60S subunits are assessed by Arabidopsis homologs of eIF6A and NMD3 of yeast cytosolic 60S maturation factors (means +/- standard error of the four fractionations of DS 1 or DS2 (Supplemental Table S4)).
