# Supplementary material for: Arabidopsis REI-LIKE proteins activate ribosome biogenesis during cold acclimation
Source: Sci Rep. 2021 Jan 28;11:2410. doi: 10.1038/s41598-021-81610-z (PMC7844247; doi:10.1038/s41598-021-81610-z)
Supplement: Supplementary file 8 — Supplementary Information 8. [file 41598_2021_81610_MOESM8_ESM.pdf]

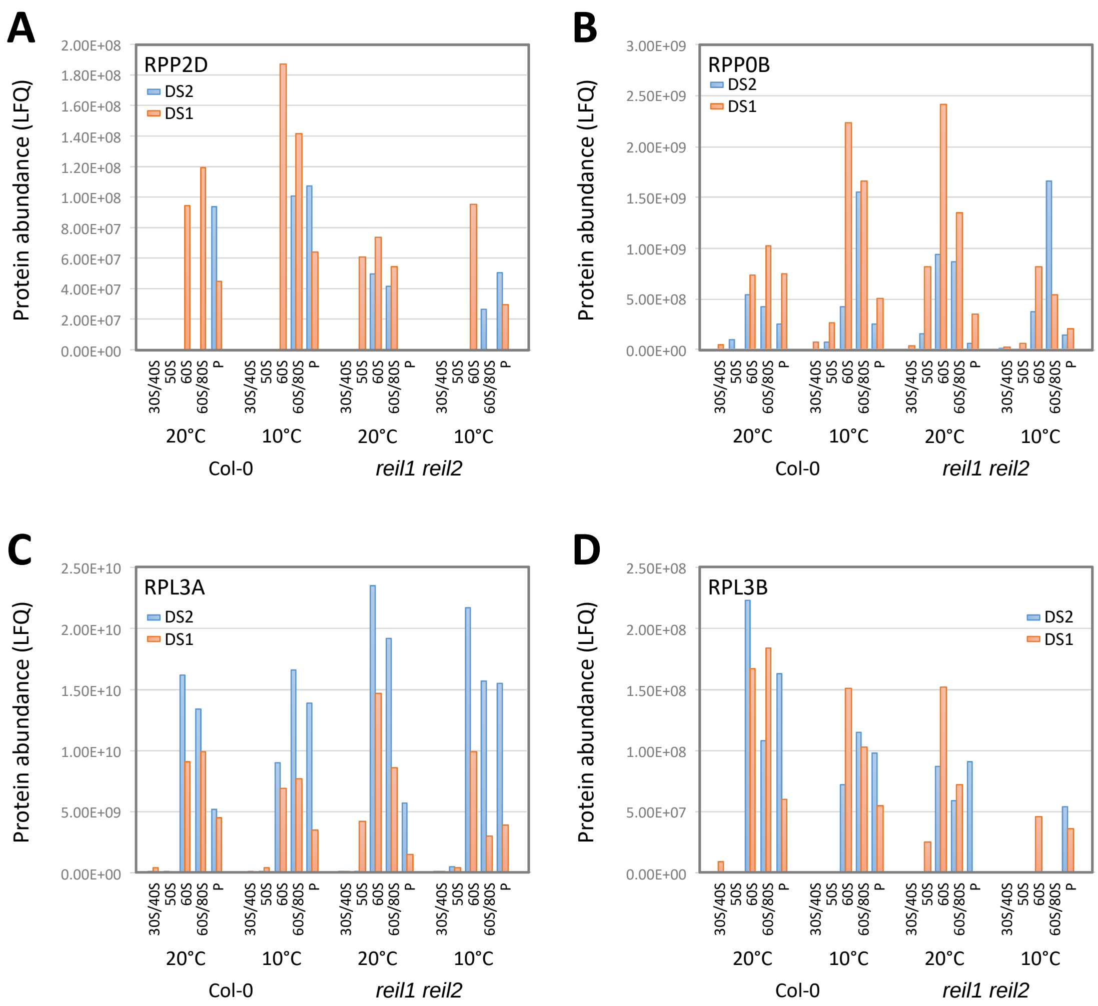
A
RPP2D
DS2
DS1
Protein abundance (LFQ)
2.00E+08
1.80E+08
1.60E+08
1.40E+08
1.20E+08
1.00E+08
8.00E+07
6.00E+07
4.00E+07
2.00E+07
0.00E+00
30S/40S
50S
60S
60S/80S
P
20°C
10°C
20°C
10°C
Col-0
reil1 reil2
B
RPP0B
DS2
DS1
3.00E+09
2.50E+09
2.00E+09
1.50E+09
1.00E+09
5.00E+08
0.00E+00
C
RPL3A
DS2
DS1
2.50E+10
2.00E+10
1.50E+10
1.00E+10
5.00E+09
0.00E+00
D
RPL3B
DS2
DS1
2.50E+08
2.00E+08
1.50E+08
1.00E+08
5.00E+07
0.00E+00

Supplemental Figure S8. Distribution analysis of RPP2D **(A)**, RPP0B **(B)**, RPL3A **(C)**, and RPL3B **(D)** across the sampled ribosome fractions and analyzed conditions. (experiment DS1, orange; experiment DS2, blue).
