# Supplementary material for: Arabidopsis REI-LIKE proteins activate ribosome biogenesis during cold acclimation
Source: Sci Rep. 2021 Jan 28;11:2410. doi: 10.1038/s41598-021-81610-z (PMC7844247; doi:10.1038/s41598-021-81610-z)
Supplement: Supplementary file 9 — Supplementary Information 9. [file 41598_2021_81610_MOESM9_ESM.pdf]

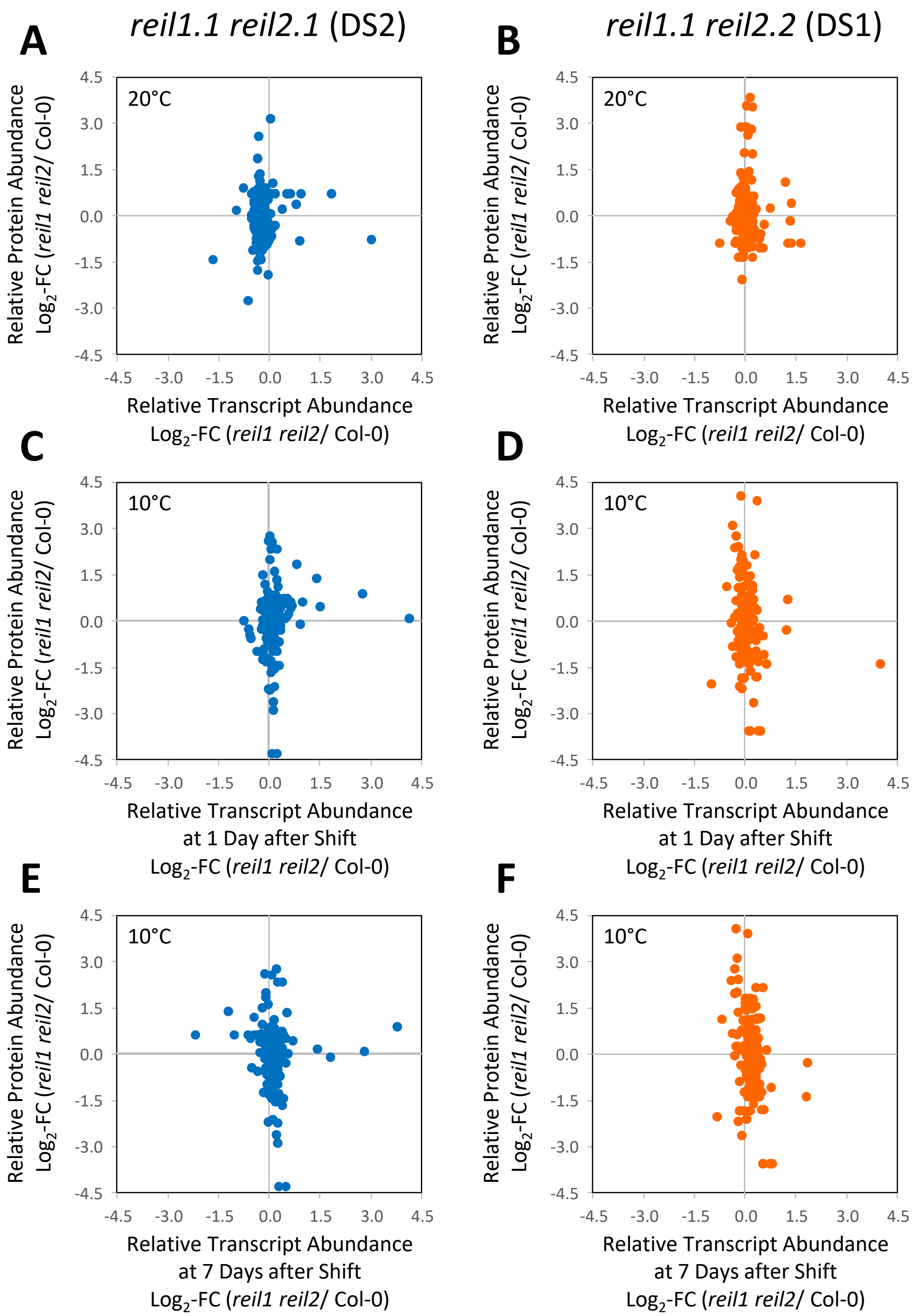

A
reil1.1 reil2.1 (DS2)
B
reil1.1 reil2.2 (DS1)
20°C
20°C
Relative Protein Abundance
Log2-FC (*reil1 reil2*/ Col-0)
Relative Transcript Abundance
Log2-FC (*reil1 reil2*/ Col-0)
C
D
10°C
10°C
Relative Transcript Abundance
at 1 Day after Shift
Log2-FC (*reil1 reil2*/ Col-0)
E
F
10°C
10°C
Relative Transcript Abundance
at 7 Days after Shift
Log2-FC (*reil1 reil2*/ Col-0)
4.5
3.0
1.5
0.0
-1.5
-3.0
-4.5

Supplemental Figure 9. Association analysis of changes in root transcript abundance and relative protein abundance in non-translating 40S and 60S (60S/80S) fractions of *reil1 reil2* mutants compared to *Arabidopsis thaliana* Col-0 wild type. Experiment DS2 (*reil1.1 reil2.1*) blue (A, C, E), experiment DS1 (*reil1.1 reil2.2*) orange (B, D, F), (A, B) relative protein and transcript abundance determined at 20°C, i.e. at day 0, prior to cold shift, (C, D) relative protein abundance determined at 7 days after shift to 10°C, transcript abundance at 1 day after shift to 10°C, (E, F) relative protein and transcript abundance determined at 7 days after shift to 10°C.
